# Pectin Films with Recovered Sunflower Waxes Produced by Electrospraying

**DOI:** 10.3390/membranes12060560

**Published:** 2022-05-28

**Authors:** Mayra C. Chalapud, Erica R. Baümler, Amalia A. Carelli, Ma. de la Paz Salgado-Cruz, Eduardo Morales-Sánchez, Minerva Rentería-Ortega, Georgina Calderón-Domínguez

**Affiliations:** 1 Departamento de Ingeniería Química, Universidad Nacional del Sur (UNS), Bahía Blanca 8000, Argentina; mchalapud@plapiqui.edu.ar (M.C.C.); ebaumler@plapiqui.edu.ar (E.R.B.); acarelli@plapiqui.edu.ar (A.A.C.); 2 Planta Piloto de Ingeniería Química—PLAPIQUI (UNS-CONICET), Bahía Blanca 8000, Argentina; 3 Escuela Nacional de Ciencias Biológicas, Instituto Politécnico Nacional, Wilfrido Massieu s/n, Unidad Profesional Adolfo López Mateos, Zacatenco, GAM, Mexico City 07738, Mexico; msalgadoc@ipn.mx; 4 CICATA—Unidad Querétaro, Instituto Politécnico Nacional, Cerro Blanco No. 141, Col. Colinas del Cimatario, Santiago de Querétaro 76090, Mexico; emoraless@ipn.mx; 5 Tecnológico Nacional de México/TES de San Felipe del Progreso, Av. Instituto Tecnológico S/N Ejido de San Felipe del Progreso, San Felipe del Progreso 50640, Mexico; minerva.ro@sfelipeprogreso.tecnm.mx

**Keywords:** electrospraying, recovered sunflower waxes, pectin films, low and high-methoxyl pectins

## Abstract

Valorization of by-products obtained from food processing has achieved an important environmental impact. In this research, sunflower wax recovered from oil refining process was incorporated to low and high-methoxyl pectin films produced by electrospraying. Film-forming solutions and wax-added electrosprayed films were physical and structurally evaluated. The addition of sunflower wax to the film-forming solutions reduces conductivity while raising surface tension and density, whereas the type of pectin had a larger impact on viscosity, with the low-methoxyl solution having the highest value. These changes in physical solution properties influenced the film characteristics, observing thicker films with lower water vapor transmission rate (WVTR) when adding wax. Micrographs obtained by scanning electron microscopy (SEM) revealed the presence of wax particles as small spherical shapes, having a good distribution through the sectional area of films. According to X-ray diffraction (XRD), atomic force microscopy (AFM) and mechanical properties analyses, the presence of wax had an impact on the degree of crystallinity, producing a more amorphous and rougher film’s structure, without affecting the elongation percentage and the tensile stress (p>0.05). These results showed that wax addition improves the physical properties of films, while the suitability of using both pectins and the electrospraying technique was demonstrated.

## 1. Introduction

Plastic films made from non-renewable resources often cause harmful effects to the environment after disposal, while the current rise in consumer needs for safe, healthy, and stable foods, besides the awareness concerning the harmful environmental effects of wastes, have increased interest in using natural materials for food packaging [1,2,3]. This situation has amplified the interest in employing edible compounds as proteins, polysaccharides, and lipids derived from diverse renewable and biodegradables sources, as well as by-products or food industry wastes as substitutes for synthetic polymers to elaborate edible films for food packaging [4,5,6]. 

Edible films and coatings act as a reinforcement of the outer protective layer of foods (peel) and as a barrier to reduce the quality deterioration process, which is promoted by the migration of water vapor, oxygen, ethylene, etc. [7,8]. Therefore, they are considered as potential systems to control mass transfer within a food matrix or between the matrix and the environment providing mechanical protection to food. Edible films are produced with biomaterials that are non-toxic, biocompatible, renewable, and always have a reasonable film formation capability. Polysaccharides such as starch, alginate, cellulose, chitosan, agar, and pectin are often less expensive and easier to obtain than other materials [3,4,5], with pectin being one of the more tested for edible packaging studies [9,10,11,12,13]. These polysaccharides constitute a friendly alternative to the environment, due to its low cost, availability, biocompatibility, edibility and biodegradability, producing films alone or needing the combination with other materials such as polymeric matrixes and lipids [6,14], due to the fact that the most of them are hydrophilic with low water barrier properties, then the incorporations of other components could improve their features [14]. 

Pectin is an anionic polysaccharide that is usually present in many primary and middle layers of the cell walls of plants [15,16], and depending on its degree of esterification (DE), which affects their gelling properties, it is classified as high-methoxyl pectin (HMPe, DE > 50%) or low-methoxyl pectin (LMPe, DE < 50%) [17,18] with molecular weights between 150 × 10^3^ and 226 × 10^3^ g/mol [19,20]. The literature on films regarding the use of pectin, or its use in combination with other materials, is not very extensive; it is more common that those studies developed using high or low-methoxyl pectin and the casting technique [17,21,22,23,24,25,26,27,28], rather than other film-forming methodologies, for example, electrospraying [12,29,30,31,32], which opens a new field of research. According to these reports, most low-methoxyl films are added with calcium salts, and both types of pectin are often combined with other wall materials such as sodium alginate, chitosan, cutin, whey protein, plasticizers (glycerol, tween or polyethene glycol), and lipids to improve film properties. Lipid materials can be incorporated during the preparation of edible films by emulsifying them with polysaccharides or proteins [33,34]. The waxes, which form part of lipid materials, improve the resistance of films to water diffusion, due to the very low content of polar groups and their high content of long-chain fatty alcohols and alkanes [9]. In this context, sunflower waxes recovered from a by-product resulting from the winterization process of sunflower oil constitute a promising material to be used for film elaboration. In this context, Chalapud et al. [35] evaluated and characterized sunflower waxes from this by-product, obtaining important data about wax composition and its potential uses, recommending its utilization to prepare films and coatings. Regarding films elaboration, the electrospraying method constitutes a novel technique commonly known as electrostatic hydrodynamic atomization process. In this technique, the emulsion or solution gets charged by electrostatic forces while passing through the nozzle connected to a high-voltage supply. These charges overcome the surface tension of the liquid and break up the liquid jet into micro-droplets [36]. These drops become electrically charged, facilitating the control of their motion (including their deviation and focus) by means of an electric field [37]. This technique has been applied on coating fruits and confectionery products [38], fresh meat using bovine gelatin [39], crackers with corn starch, methylcellulose and soybean oil [40] and fresh strawberries using alginate [41]. In previous studies of the research group [12,29,31,37], the electrospraying technique has been optimized by performing tests, developing, and evaluating physical and structural characteristics of films based on zein [37] and pectin [12,29,31]. The results obtained from these research works were compared with those obtained by the casting method, indicating good properties of the films obtained by the electrospraying technique. In a previous work [10], edible films with three concentrations of low-methoxyl pectin and three wax proportions of recovered sunflower waxes were elaborated by casting method. In the present study, in order to continue exploring the use of waxes recovered from sunflower oil refining process into production of films, the investigation was extended to the use of high-methoxyl pectin with a novel technique such as electrospraying method and carrying out other characterization techniques.

The aim of this work was to study the feasibility of producing electrosprayed films with low and high-methoxyl pectin added with recovered sunflower wax, evaluating the structure, composition, water resistance, and mechanical properties of the films as a result of the different materials used.

## 2. Materials and Methods

### 2.1. Materials

High-methoxyl pectin (HM) from citrus peel (P9135, Sigma-Aldrich, Mexico) with a galacturonic acid content ≥74% (dried basis) and methoxy groups ≥6.7% (dried basis), partly amidated low-methoxyl citrus pectin (LM) (GENU PECTIN type LM 104 AS, CP Kelco, Argentina) with esterification degree approximately 27%, recovered sunflower wax (SFW), previously obtained of waste material from sunflower oil refining process (winterization stage) and characterized [35] were used for films elaboration. Glycerol was employed as a plasticizer (G5516, Sigma-Aldrich, Mexico) and Tween 20 as surfactant (P1379, Sigma-Aldrich, Mexico). Calcium Chloride (CaCl2) and Sodium Bromide (NaBr) were obtained from J.T. Backer Inc. (Phillipsburg, NJ, USA) (98–99% purity, analytical grade reagent). 

### 2.2. Elaboration of Solutions and Emulsions

Control solutions (without wax) were prepared by hydrating and stirring pectin (HM or LM) with distilled water at 2% *w/w* to complete dissolution at room temperature. Glycerol was added at 50 g/100 g pectin and Tween 20 in a ratio 1:10 (*w/w*) (Tween 20:pectin) and the mixture was stirred for 10 min. Pectin and sunflower wax emulsions were performed according to the procedure described by Chalapud et al. [33] with some modifications. The resulting solutions—low or high-methoxyl pectins, glycerol and surfactant—were heated at 85 °C (above the melting point of the wax) maintaining the temperature by a water bath, and then the SFW at 0.2 g/g pectin was added. This SFW ratio was selected based on data reported by Chalapud et al. [10]. All emulsions were homogenized for 10 min using an Ultra Turrax (T25 Basic IKA Labortechnik) at 13,000 rpm at 85 °C. For emulsions with LM pectin, calcium chloride was added at 1 g/100 g pectin.

Physicochemical properties of pectin control solutions and sunflower wax emulsions such as conductivity, viscosity, density and superficial tension were evaluated as quality parameters.

### 2.3. Density, Refractive Index and Surface Tension

Density and refractive index of solutions and emulsions was evaluated according to 962.37 and 990.35 methods of the A.O.A.C. International [42]. Surface tension was performed using a Krüss K6 tensiometer (Krüss Company, Germany) according to the Du Noüy ring method with a platinum ring, following the manufacturer procedure. Samples were analyzed in triplicate.

### 2.4. Electric Conductivity (σ)

Conductivity determination was performed following two-point resistivity technique [43] using a multimeter (LCR HiTester, HIOKI-3532-50; Nagano, Japan), which recorded resistivity (R) data at three different frequencies (0.1, 1 and 10 kHz). Electric conductivity values of solutions and emulsions were calculated by Equation (1):

(1) $$\sigma =\left(\frac{1}{R}\right)*\left(\frac{L}{A}\right)$$

where *σ* (kΩ^−1^ m^−1^) is conductivity, *R* (kΩ) the resistivity, *L* (m) is the distance between two electrodes and *A* (m^2^) is cross-sectional area of cell.

### 2.5. Viscosity

This parameter was performed using a HR-3 Discovery Hybrid Rheometer (TA Instruments, New Castle DE, USA) and a concentric cylinder geometry with a cup (diameter = 30.36 mm) and rotor (diameter = 28.02 mm, and height = 42.2 mm). Volume sample was 23 mL and the tests were performed by duplicate at 25 °C without pre-shear. Share rate range was between 0.01–100 s^−1^.

### 2.6. Electrosprying

Electrospraying technique was followed using the equipment and procedure described by Gaona Sánchez et al. [29] with some modifications according to the preliminary test conditions obtained. In a syringe pump, 10 mL of samples solutions were placed. The syringe was connected to the positive high voltage power supply (0–30 kV DC, Model 30A24-P4 Brand Ultravolt, NY, USA) obtaining a constant injection flow (3 mL/h) and being moved by a lineal actuator at 0.667 cm/min. The negative electrode was connected to a rotary drum (rotation speed of drum = 2.4 rpm), which was placed at 50 cm of the injection point. On the opposite side to the injected flow, a hot air dryer (150 °C, 51.4 L/h, Steren Hot Air Station CAU-280, China) was placed at 2.5 cm from drum to preheat the collector (60 °C) and to help drying the pectin film. An electric voltage of 7.8 ± 0.5 kV was applied. All samples were processed at these conditions to obtain homogeneous films free of fibers and electric arcs. Films of approximately 15 × 8 cm^2^ were obtained and were stored at room temperature inside a desiccator with a saturated solution of NaBr at 57% relative humidity (RH) before testing.

### 2.7. Films Characterization 

#### 2.7.1. Film Thickness

Thickness was measured at five sites of the films (one in the center and four on the periphery) by using a digital micrometer model ID-C112EXB (Mitutoyo Corp., Japan). The average value was used for calculations of mechanical and permeability properties.

#### 2.7.2. Scanning Electron Microscopy (SEM) 

Structure of top surface and cross section of films was examined by SEM. Samples were covered with a thin layer of gold in a Denton Vacuum Desk II. The micrographs were obtained with a scanning electron microscope JEOL JSM 6460 LV (JEOL Ltd., Japan) at an acceleration voltage of 15 kV and 1500x magnification. 

#### 2.7.3. Atomic Force Microscopy (AFM)

Topographic AFM images in three dimensions and at real-time resolution were obtained by an atomic force microscope (diMultimode V, Veeco, Santa Barbara, CA, USA) with a resonance frequency of 286–362 kHz and a force constant of 20–80 Nm^−1^., reporting images roughness (Rq, Ra). For this evaluation, for each sample, sections of 0.5 cm × 0.5 cm were cut and two areas of 10 × 10 μm^2^ were scanned at a speed of 1 Hz and at a resolution of 256 × 256 pixels. The surface roughness was measured from images calculating the root square deviation of the heights (Rq) (Equation (2)) and the arithmetic mean of the absolute values of the height deviations (Ra) (Equation (3)) using the Nano Scope Analysis 1.5 program (Veeco, Santa Barbara, CA, USA) and applying a flattening process (one degree). Determinations were performed in triplicate.

(2) $$Rq=\sqrt{\frac{\sum^{}{\left(Z_{i}\right)}^{2}}{N}}$$

(3) $$Ra=\frac{1}{N}\sum_{i=1}^{N}\left|Z_{i}\right|$$

where *Z_i_* is the height deviation from the mean of the heights, and *N* is the number of points in the image. 

#### 2.7.4. X-ray Diffraction

X-ray diffractograms were obtained by a Rigaku Miniflex 600 powder diffractometer (CuK radiation, λ = 0.54°A) operating at 45 kV and 40 mA. The scanning regions were in a 2θ range of 3°–35°. The degree of crystallinity (%) of films was calculated using Equation (4). The separation and integration of the areas under the crystalline and amorphous X-Ray diffraction peaks was performed as recommended by Ribotta et al. [44]. The software PeakFit version 4.12 (SYSTAT Software) was used for this purpose. Determinations were performed in triplicate.

(4) $$Degree\;of\;Crystallinity=\left(\frac{A}{A+B}\right)*100$$

where *A* is the integrated intensity of the crystalline phase and (*A* + *B*) is the total area of the diffractogram.

#### 2.7.5. Water Vapor Permeability (WVP)

WVP of films was determined according to the methodology proposed by Cazón et al. [45] based on the ASTM Standard E96/E96M-05 [46] with some modifications, and following the methodology reported by Valdespino-León et al. [12]. Films samples were cut into 3.3 cm diameter circles and placed on the top of the permeation cell that contained water (100% RH). The permeation cell was placed in an environment with anhydrous silica gel (0% RH) at controlled temperature (30 °C). The weight of the cell was recorded every 30 s in an analytical balance (Denver Instruments model TP-214) for 24 h, obtaining the water vapor transmission rate (WVTR, gs^−1^m^−2^) by dividing this ratio (g/s) by the area of tested film. WVP was calculated according to the combined Fick and Henry laws for gas diffusion through films, using Equation (5), where x is the film thickness (m) and ∆P corresponds to the vapor pressure difference inside the system (4246.9 Pa). The measurements were performed in triplicate.

(5) $$WVP=WVTR.\frac{x}{\Delta P}$$

#### 2.7.6. Mechanical Properties

Mechanical properties (tensile strength (TS) and elongation at break (%E)) were evaluated according to the ASTM Standard D882-09 [47], using a texture meter (Texture analyzer CT3, Brookfield ™, USA) and its software (TexturePro CV V1.6), following the methodology reported by Valdespino-León et al. [12]. Samples were cut into rectangles of 2.5 cm wide and 12.5 cm long and placed in the instrument’s dual grip assembly (TA-DGA accessory). The test conditions were performed at an activation load of 450 g, a speed of 0.3 mm/s, and a return speed of 4.5 mm/s with a 4500 g load cell. TS (MPa) was calculated (Equation (6)) by dividing maximum force load (N) at the rupture point by film cross-section (mm^2^), while %E (Equation (7)) by the film stripe length changes during elongation, both methodologies according to Cazón et al. [45]. Young’s module was also evaluated by the TS/E ratio (Equation (8)). Data were obtained in triplicate.

(6) $$TS=\frac{F}{L*x}$$

where *L* is the film width (mm), and *x* is the thickness (mm).

(7) $$\%E=\frac{\Delta L}{L}\times \;100=\frac{L_{f}-L_{i}}{L_{i}}\;\times \;100$$

where *L_f_* is the length of film at break and *L_i_* is the final length.

(8) $$Y=\frac{F/A}{\Delta L/L}=\frac{TS}{E}$$

#### 2.7.7. Raman Spectroscopy

Raman analysis was performed using a LabRam HR800 (Horiba Jobin Yvon, Miyanohigashi, Kyoto, Japan) coupled to an Olympus BX 41 microscope with a 100× objective and a Peltier-cooled charged coupled device detector and three excitation lines: 532 nm, 633 nm and 785 nm with output power of 43.4 mW, 86.3 mW and 56.7 mW, respectively. Raman spectra were recorded using 400 lines/mm and an emission laser of 785 nm. Calibration was determined using the 520.5 cm^−1^ silicon line. The measurements were performed at wave numbers from 100 cm^−1^ to 3200 cm^−1^ at room temperature. The spectra were recorded by applying an exposure time of 4–60 s and scanning the sample 10 to 20 times. The acquired spectra were managed by LabSpec software (Horiba Jobin Yvon) and edited using Spectragryph program version 1.1.2. The measurements were performed in triplicate.

#### 2.7.8. Statistical Analysis

Significant differences were determined using one-way ANOVA comparing all samples simultaneously. The type of pectin (high- or low-methoxyl), and the presence of wax were the variables studied. Each response (columns) was statistically analyzed and labeled with different lowercase letters provided by Fisher Test (α = 0.05) where values followed by different letters indicate significant differences (*p* < 0.05). For statistical analysis, InfoStat statistical analysis software, Version 2011 was used. 

## 3. Results and Discussion

### 3.1. Film-Forming Solutions Characterization

Table 1 shows the physicochemical properties of pectin control solutions (HMsol and LMsol) and sunflower wax emulsions (SF-HMem and SFW-LMem) used to elaborate the films. The conductivity did not follow a homogenous behavior, observing the highest value for LMsol, while the wax addition to this material sharply decreased this value. The presence of pectin (HM or LM) in solutions and emulsions reduced the surface tension of water (72 mN/m at 25 °C) to 37.8–43.6 mN·m^−1^. This reduction was reported by Yang et al. [48] and Yapo et al. [49], who studied the emulsified properties of pomegranate peel pectin and surface properties of beet pulp pectin, respectively. Moreover, HMsol and SFW-HMem presented conductivity values in the same magnitude order, with LMsol showed a significative increase. This result is attributable to the fact that HM pectins have a lower charge density and high molecular weight ions with less mobility than LM pectins, which influence the conductivity values [50]. Density was affected by the presence of wax, slightly increasing this parameter by its addition and having similar values to those reported by Gaona Sánchez et al. [29] for high-methoxyl pectin solutions. Viscosity changed in function of the type of pectin, having low-methoxyl materials (LMsol and SFW-LMem) the highest values (*p* < 0,05), and following a shear thinning behavior as indicated by the flow index, whose values are further away from 1 than those found for solutions and emulsions with high-methoxyl pectin (HMsol and SFW-HMem), which present a behavior close to Newtonian. In this regard, Morris et al. [51] found an intrinsic viscosity reduction of pectin solutions as DE decreases; however, in this work, the differences in viscosity values between types of pectins can be attributed to the addition of calcium to LM pectin mixtures to promote gelling, which caused a significant increase in the viscosity of solutions and emulsions elaborated with LM pectin. Moreover, despite the wax addition, the viscosity of emulsions did not change significantly (*p* > 0.05).

### 3.2. Films Characterization

Table 2 shows the thickness and water vapor permeability (WVTR, WVP) of pectin films added or not with sunflower wax and prepared by electrospraying method.

These results showed that SFW-HM and SFW-LM presented higher thickness values than control samples, without a significant effect due to the type of pectin (*p* > 0.05). These results are related with the increase in density of sunflower wax added emulsions as compared to control solutions without wax inclusion.

Regarding WVTR of films, these values decreased with wax addition, finding significant differences (*p* < 0.05) when compared to non-wax added films. These results show that sunflower wax offered an important barrier to water vapor transference mainly due to its hydrophobic character caused by their high content of long-chain fatty alcohols and alkanes [32,52], wherein the waxes are considered as one of the most efficient substances in the water vapor transference reduction [53,54,55]. Likewise, dispersion of hydrophobic compounds may increase the hydrophobicity, leading to a decrease in water vapor diffusion rates [56], but a homogenous distribution in the support matrix is needed [11]. Zhang et al. [57], Brzoska et al. [58] and Fabra et al. [59] discovered a similar decrease in WVTR by incorporating beeswax into chitosan and sodium caseinate films, respectively, demonstrating the high efficacy of beeswax as a vapor barrier, especially when the wax concentration increase. Although sunflower wax incorporation influenced the decreasing of the water vapor transmission rate (WVTR), WVP values were affected by its positive correlation with thickness, which, as expected, increased with the wax presence (Table 2). Similar behaviors were reported by Debeaufort and Voilley [5], and Martin-Polo et al. [60], who noted an increase in WVP when thickness increased due to film discontinuities in paraffin-based and cellophane films, respectively. Giancone et al. [61] reported that increased thickness of high-methoxyl pectin films raised their density, and that there was a positive correlation between WVP and film density. 

The values of WVTR and WVP for LM pectin films were slightly lower than those elaborated using HM. These differences can be related to the mechanisms of pectin gelation. It has been reported that noncovalent forces, as hydrophobic interactions are responsible for gel formation in high methoxyl pectins [62], while low methoxyl pectins form gels by interactions between Ca2+ ions and carboxyl groups, producing a structure named “egg box” [17,63]. The gels structure produced by low-methoxyl pectins could be more stable to moisture than those formed by high-methoxyl, adding “apparent tortuosity” for water molecules migration.

### 3.3. SEM Microscopy

**Figure 1:**
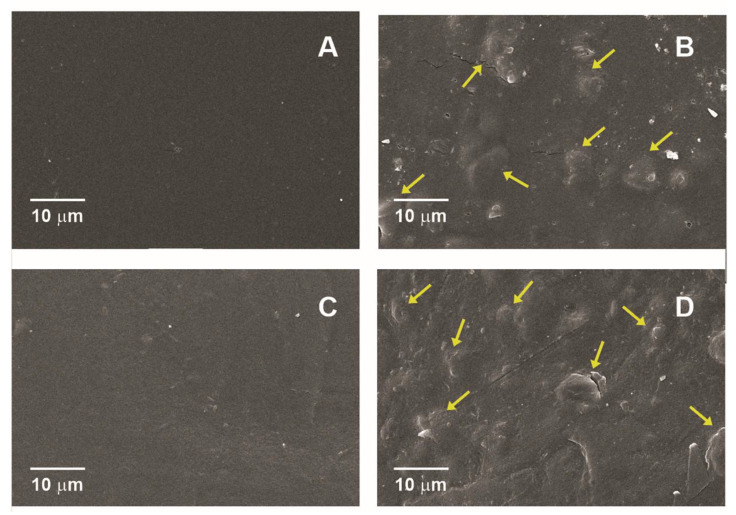
Top section of films. (**A**) control high-methoxyl pectin film (HM); (**B**) high-methoxyl pectin film with sunflower wax addition (SFW-HM); (**C**) control low-methoxyl pectin film (LM); (**D**) low-methoxyl pectin film with sunflower wax addition (SFW-LM). Arrows indicate sunflower wax bodies in pectin films.

Figure 1 shows the top section micrographs of pectin films (added or not with sunflower wax), obtained by SEM microscopy.

Micrographs of the top surface of films revealed the presence of a support matrix corresponding to pectin (HM or LM) and sunflower waxes as small spherical shapes (arrows) that produce an irregular area with some cracks and areas that correspond to pectin matrix. These results were similar to those obtained in a previous work by Chalapud et al. [10], who used the casting method to elaborate low-methoxyl pectin films with the inclusion of sunflower wax. On these SEM images an immiscibility between the hydrophilic components (pectins) and hydrophobic materials (waxes) is observed, having a fine particle entrapment on the film surface [64]. Besides, the fractal disposition of lipidic particles on the film surface due to van der Waals forces [65] and the layers with which a film is produced can develop more irregularities [66]. It is also observed from these micrographs that top surfaces of HM and LM were more uniform and homogenous than those added with sunflower wax. Similar results in films structure were presented by Galus et al. [67], and Meerasri and Sothornvit [68] for high-methoxyl pectin edible films, and Bierhalz et al. [69] who used low-methoxyl pectin crosslinked with calcium ions to obtain films. 

**Figure 2:**
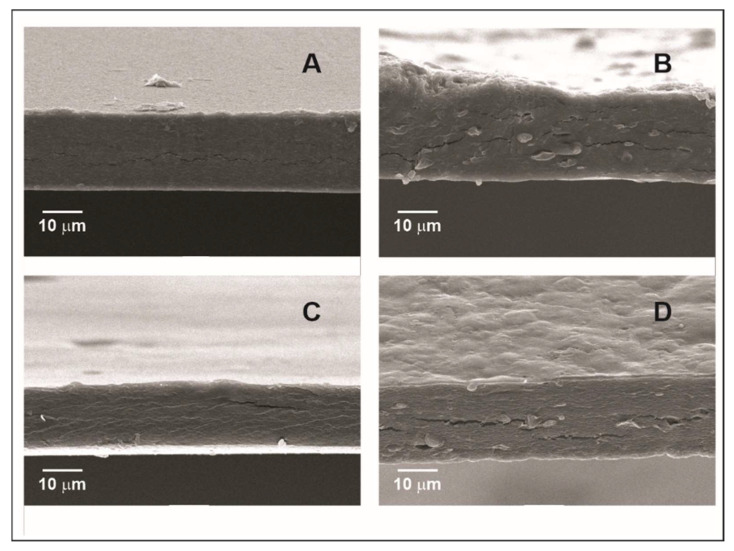
Cross section of films. (**A**) control high-methoxyl pectin film (HM); (**B**) high-methoxyl pectin film with sunflower wax addition (SFW-HM); (**C**) control low-methoxyl pectin, (**D**) low-methoxyl pectin film with sunflower wax addition (SFW-LM).

Images of cross-sectional area of films (Figure 2) showed a smoother surface and lower thickness of HM and LM films than SFW-HM and SFW-LM, in which the presence of small globular possibly wax bodies distributed and dispersed throughout the film is observed. Unlike previous work in which the films were elaborate by casting [10], no destabilization phenomena of the emulsions such as creaming was observed in these cross-sectional images. The different behavior can be attributed to the shorter drying time of the films made by electrospraying, because the emulsion was atomized and then dried in the rotary drum with the hot air dryer. Moreover, despite the existence of cracks around the wax bodies, their shallow depth, hydrophobic character and dispersion of wax inside the pectin network structure attenuated the effect of increased water molecule diffusion between the two sides of the film. 

### 3.4. X-ray Diffraction

**Figure 3:**
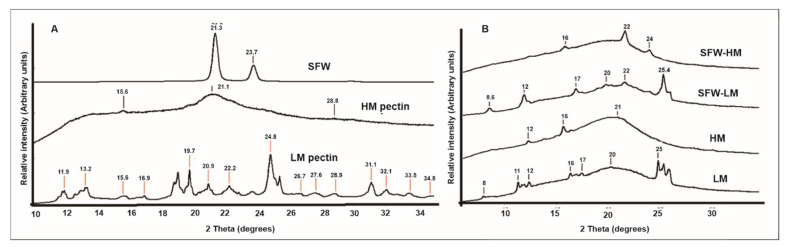
XRD patterns of raw materials (**A**) and control pectin films and sunflower wax pectin films (**B**) HM: Control high-methoxyl pectin film; SFW-HM: high-methoxyl pectin with sunflower wax addition; LM: Control low-methoxyl pectin film; SFW-LM: low-methoxyl pectin film with sunflower wax addition.

In Figure 3, X-ray diffraction patterns of pectin films (Figure 3A) and raw materials (Figure 3B) are shown.

According to X-ray patterns of raw materials (Figure 3A), HM pectin presented two mean peaks at 21° and 15.6°. Similar patterns were reported by Valdespino-León et al. [12] and Lutz et al. [70]. HM pectin has a more amorphous structure compared to LM pectin, behavior that is also observed in the film X-ray diffraction curves (Figure 3B), where main peaks at 12°, 16° and 21° for HM films and at ~11–12°, 16–17°, 20° and 25° for LM films are presented and confirmed by the degree of crystallinity results (Table 3). Mean peaks found in HM pectin were conserved in control and sunflower wax HM films, while most of the peaks usually observed in LM pectin raw material (Figure 3A) at 13.2, 15.6, 20.9 and 31.1° are reported to disappear with crosslinking treatment with Ca2+ during film formation, implying that the structure created by junction zones lost some of its order and increased the number of amorphous points in the molecular structure due to the presence of Ca2+ and their X-ray absorption capacity. [27]. On the other hand, recovered sunflower wax consisted in long chain esters with 40–60 carbon atoms [35] and presented, in solid state, an ordered structure with two peaks at 21.3 and 23.7° (Figure 3A), which correspond to CH_2_ groups within hydrocarbon chains that integrate into the crystalline configuration [71]. When the sunflower waxes are melted, the crystalline structure is disordered, producing a conformational change and fitting the polar ester heads of sunflower wax in the amorphous phase [72]. Therefore, addition of melted sunflower wax to pectin films had an impact on the degree of crystallinity reduction in SFW-HM and SFW-LM films (Table 3), promoting a different structure, decreasing crystallinity and generating a more amorphous structure that appear as a wide band centered at 22°, mainly for the high-methoxyl pectin films (*p* < 0.05). On the other hand, the high degree of crystallinity presented by LM films (Table 3) is connected to their WVP reduction (Table 2), due to the decrease in molecular mobility and water molecules accessibility [12,27]. 

### 3.5. Atomic Force Microscopy (AFM)

**Figure 4:**
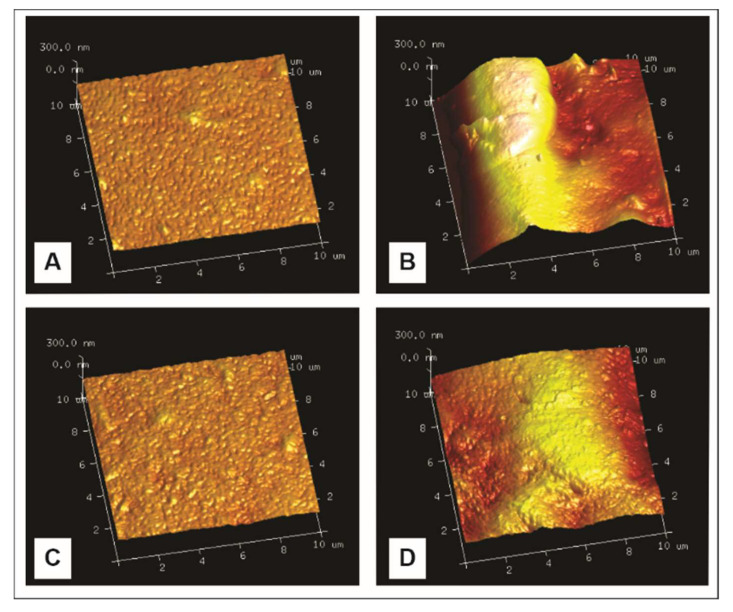
AFM height 3D images of pectin films. (**A**) control high-methoxyl pectin film (HM); (**B**) high-methoxyl pectin film with sunflower wax addition (SFW-HM); (**C**) control low-methoxyl pectin, (**D**) low-methoxyl pectin film with sunflower wax addition (SFW-LM).

Rq and Ra values used to evaluate the surface roughness of films are presented in Table 3. The morphologies are also observed in AFM height images (Figure 4). Control HM films had significantly lower Rq and Ra values (*p* < 0.05) than control LM films, which could be attributed to changes in the gelling mechanisms of each type of pectin, resulting in a less homogenous surface in those films elaborated with LM pectin due to the creation of junction zones. For control LM pectin films, these results are associated with a high degree of crystallinity and network structure revealed by X-ray diffraction (Table 3). Rq and Ra values for control HM films were lower than those obtained by Valdespino-León et al. [12] and Gaona Sánchez et al. [29]. Besides, as expected, and reinforcing SEM micrographs, both HM and LM films presented smoother surfaces (Table 3) with lower roughness Rq and Ra values (*p* < 0.05) than SFW-HM an SFW-LM. Eghbal et al. [73] reported that the control LM pectin film had a smooth and homogeneous structure that those with sodium caseinate addition. Although destabilization phenomena of emulsions were not visible in cross sectional SEM images of films (Figure 2) due to the good distribution of globular wax bodies throughout the film (as mentioned above), the presence of wax on the top of the surface of films, which increases their thickness, is evidenced by the high values of surface topography of sunflower wax pectin films (Table 3). In this sense, the immiscibility of material components, the disposition of lipidic particles due to interactions and their entrapment on support matrix led non-homogeneity surfaces on films [74].

### 3.6. Mechanical Properties

Table 4 shows the mechanical properties of control and sunflower wax pectin films. Wax incorporation of LM films (SFW-LM) produced a higher elasticity (%E), while HM films (SFW-HM) behaved the opposite way. Although no significant differences were found in %E values (*p* > 0.05), according to Campos et al. [75] and Harnkarnsujarit et al. [76], as the structural cohesiveness and crystallinity degree of films increases, their flexibility reduces, and therefore their elasticity. Then, comparing control films, those elaborated with LM pectin had a more crystalline (Table 3) and cohesive structure compared to control HM film. This behavior was not evidenced with wax addition to pectin films, since the low crystallinity in SFW-HM films did not alter their elongation values (*p* > 0.05). However, in SFW-LM films, a slightly increase in %E was observed, possibly due to interactions generated by the carboxyl groups of LM pectin [77].

Regarding tensile stress at break, films elaborated with high-methoxyl pectin and sunflower wax (SFW-HM) presented the highest TS value, followed by HM films, which can be related to the lower degree of crystallinity presented by SFW-HM (Table 3). LM films, on the other hand, had a more crystalline structure (Figure 3A), and the crosslinked network produced and combined with sunflower wax inclusion resulted in a 25–40% reduction in the maximum tensile stress the material could support. This TS reduction was reported by Harnkarnsujarit et al. [76], who obtained a decrease in tensile strength in films with high crystallinity after adding microcrystalline cellulose gums. This fact also is reflected in the decrease in elastic modulus (Y) in LM films, indicating that their crystalline tie points do not contribute to the stiffness increase. For both types of pectin, although the wax addition resulted in a not significant increasing of TS values (*p* > 0.05) and a not significant variation of Y (*p* > 0.05), the dispersion of wax bodies serves as focus areas, decreasing chain mobility, promoting tensile strength and acting in stiffness of films [34,78]. In general, HM films exhibited higher TS and lower %E, being more resistant and less flexible with wax addition, while LM films presented TS values lower with %E that increased with sunflower wax incorporation, what makes the film more flexible and less mechanical resistant. These results are similar to those reported to Rambabu et al. [79], who found this inverse relationship between TS and %E in chitosan films with addition of mango leaf extract. Although different authors reported that the addition of waxes results in films with high tensile strength improving the mechanical properties of thermoplastic mixtures [80] and cellulose films [81], it is possible that the immiscibility between the hydrophobic and hydrophilic components of film, as well as the lack of homogeneity distribution of lipid particles into the pectin network has altered the polymer adhesion and has impacted their mechanical properties [82].

### 3.7. Raman Spectroscopy

**Figure 5:**
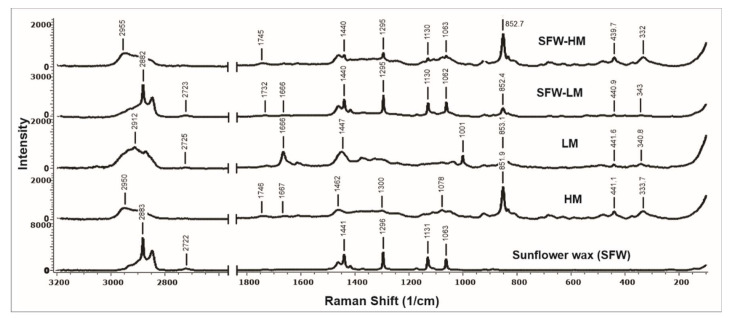
Raman spectrograms of films. Recovered sunflower wax (SFW); (**A**) control high-methoxyl pectin film (HM); high-methoxyl pectin film with sunflower wax addition (SFW-HM); control low-methoxyl pectin and low-methoxyl pectin film with sunflower wax addition (SFW-LM).

The resulting Raman spectra of sunflower wax and films obtained by electrospraying are presented in Figure 5. Sunflower waxes are esters derived from fatty alcohols and fatty acids, whose vibrational bands at 2883 cm^−1^ corresponds to asymmetric CH_2_ stretch and 2849 cm^−1^ to symmetric CH_2_ and CH_3_-CH stretching [83,84]. In addition, Raman vibrations in sunflower wax at 1063 and 1131 cm^−1^ could be assigned to C-O and C-C bonds, respectively [84], and at 1296 and 1441 cm^−1^ vibrations allocated to CH_2_ [85]. As expected, these Raman bands, with lower intensity, were also found in SFW-HM and SFW-LM films. 

The Raman spectra of films presented some important peaks in 3000–2700 cm^−1^ region. Vibrations at ~2950 cm^−1^ are assigned to the C-H bonds of the pyranoid ring carbons, which is associated with the presence of galacturonic acid in pectins [31,86,87,88]. Notice that this peak was more intense in high-methoxyl pectin films. Raman spectrum of HM and SFW-HM presented a weak peak at 1745 cm^−1^ and LM and SFW-LM at 1732 cm^−1^, which was assigned to C = O stretching of COOH [86]. Additionally, in Raman spectra, vibrations corresponding to amide I band at 1666 cm^−1^ were found in films elaborated with LM pectin (raw material partly amidated) [89]. A Raman band marker at around 852 cm^−1^ and a slight wavenumber shift between HM and LM pectins were reported by Szymańska-Chargot et al. [88]. This Raman vibration reflects the α-glycosidic bonds representative of polysaccharides [87,90], with this band shifted at around 853 cm^−1^ for LM films and 851 cm^−1^ for HM films (Figure 5), suggesting differences in pectin distribution by low or high esterification levels. Furthermore, ester groups for C-O-C bonds are indicated by peaks ranging from 439 to 441 cm^−1^ [91], which constitutes a dominant feature of pectins. 

## 4. Conclusions

Pectin films with sunflower wax addition produced by electrospraying were elaborated. Solutions and emulsions were prepared and characterized. Spherical shapes along the cross section of films showing a fine entrapment of sunflower waxes non homogenously distributed into the support matrix with small voids presence and without visual destabilization phenomena were observed by SEM. AFM analysis showed a smother surface in control pectin films and a high roughness in SFW films, due to the immiscibility of lipidic particles into pectin matrix. Despite the presence of voids, and the more crystalline structure in LM films, the addition of sunflower waxes to pectin films resulted in a decrease in molecular mobility, and water molecule accessibility, and therefore reduced the rate of water vapor transmission, demonstrating that waxes are an effective water vapor flux barrier, and the formed network had good water resistance. Films elaborated with HM pectin resulted in being more resistant, less flexible and stiffer than those films from LM pectin; however, the non-homogeneity of lipidic material distribution could influence the mechanical properties of films. Raman studies indicated that the electrospraying technique did not cause structural changes in the films or chemical interactions and mean bonds and groups from raw materials were conserved. This study showed that useful results for pectin film elaboration with sunflower waxes addition by electrospraying technique presents good functional properties.

## Figures and Tables

**Table 1:** Physical properties of pectin solutions and sunflower wax pectin emulsions.

Samples	Conductivity [μS/cm]	Surface Tension [mN·m^−1^]	Density [g·mL^−1^]	Viscosity [Pa·s]	Flow Index n
HM_sol_	858.3 ± 28.3 ^b^	37.8 ± 0.5 ^a^	1.0164 ± 0.0007 ^a^	0.0578 ± 0.0001 ^a^	0.9834 ± 0.0004 ^c^
LM_sol_	1012.3 ± 42.0 ^d^	38.8 ± 0.3 ^ab^	1.0158 ± 0.0005 ^a^	0.2254 ± 0.0519 ^b^	0.7408 ± 0.0200 ^a^
SFW-HM_em_	940.7 ± 31.6 ^c^	43.6 ± 0.4 ^c^	1.0280 ± 0.0016 ^b^	0.0976 ± 0.0021 ^a^	0.9788 ± 0.0006 ^c^
SFW-LM_em_	64.9 ± 1.2 ^a^	39.9 ± 0.8 ^b^	1.0273 ± 0.0023 ^b^	0.2236 ± 0.0243 ^b^	0.8066 ± 0.0172 ^b^

HM_sol_: high-methoxyl pectin solutions; LM_sol_: low-methoxyl pectin solutions; SFW-HM_em_: high-methoxyl pectin emulsions (with wax addition); SFW-LM_em_: high-methoxyl pectin emulsions (with wax addition); n: flow behavior index. The results are the average of at least three independent simples. Values in the same column followed by different lowercase letters (a, b, c) are significantly different (*p* < 0.05) by the Fisher Test.

**Table 2:** Thickness, water vapor transmission rate (WVTR) and water vapor permeability (WVP) of high and low-methoxyl pectin films with and without sunflower wax addition.

Film	Thickness (mm)	WVTR × 10^3^ (g/h cm^2^)	WVP × 10^4^ (g mm/KPa h cm^2^)
HM	0.023 ± 0.003 ^ab^	9.54 ± 1.24 ^b^	0.51 ± 0.03 ^b^
LM	0.018 ± 0.003 ^a^	8.29 ± 0.46 ^b^	0.36 ± 0.04 ^a^
SFW-HM	0.031 ± 0.007 ^c^	6.60 ± 0.40 ^a^	0.41 ± 0.03 ^a^
SFW-LM	0.026 ± 0.006 ^bc^	6.43 ± 0.26 ^a^	0.40 ± 0.01 ^a^

HM: control high-methoxyl pectin films; LM: control low-methoxyl pectin films; SFW-HM: high-methoxyl pectin films with sunflower wax addition; SFW-LM: low-methoxyl pectin films with sunflower wax addition. Mean values ± standard deviation were calculated from at least three independent repetitions (n = 3). Values in the same column followed by different lowercase letters (a, b, c) are significantly different (*p* < 0.05) by the Fisher Test.

**Table 3:** Degree of crystallinity (%) and roughness (Rq, Ra) of control and sunflower wax pectin films.

Film	Degree of Crystallinity (%)	Rq (nm)	Ra (nm)
HM	8.6 ± 2.0 ^b^	10.8 ± 1.5 ^a^	7.6 ± 1.0 ^a^
LM	10.4 ± 2.6 ^b^	15.5 ± 2.1 ^b^	12.5 ± 1.7 ^b^
SFW-HM	1.7 ± 0.2 ^a^	59.0 ± 4.3 ^c^	43.9 ± 1.9 ^c^
SFW-LM	9.1 ± 0.4 ^b^	56.7 ± 4.5 ^c^	45.3 ± 4.2 ^c^

HM: control high-methoxyl pectin films; LM: control low-methoxyl pectin films; SFW-HM: high-methoxyl pectin films with sunflower wax addition; SFW-LM: low-methoxyl pectin films with sunflower wax addition. Mean values ± standard deviation of at least three independent repetitions (n = 3). Values in the same column followed by different letters (a, b, c) are significantly different (*p* < 0.05) by the Fisher Test.

**Table 4:** Elongation percentage (%E), tensile stress at break (TS) and elastic modulus (Y) of control and sunflower wax pectin films.

Film	%E	TS (MPa)	Y (MPa)
HM	6.12 ± 1.62 ^a^	9.08 ± 1.10 ^ab^	202.54 ± 8.56 ^c^
LM	5.07 ± 1.72 ^a^	6.84 ± 0.50 ^a^	171.98 ± 3.84 ^a^
SFW-HM	5.53 ± 1.25 ^a^	11.68 ± 2.20 ^b^	198.75 ± 1.35 ^bc^
SFW-LM	7.53 ± 0.34 ^a^	6.99 ± 0.56 ^a^	186.77 ± 6.13 ^ab^

HM: control high-methoxyl pectin films; LM: control low-methoxyl pectin films; SFW-HM: high-methoxyl pectin films with sunflower wax addition; SFW-LM: low-methoxyl pectin films with sunflower wax addition. Mean values ± standard deviation of at least three independent repetitions (n = 3). Values in the same column followed by different letters (a, b, c) are significantly different (*p* < 0.05) by the Fisher Test.

## Data Availability

Under request.
